# Protocol: A mixed-methods study to evaluate implementation and outcomes of U.S. state telemental health policy expansion during the COVID-19 pandemic

**DOI:** 10.1371/journal.pone.0312665

**Published:** 2024-11-21

**Authors:** Lucinda B. Leung, Jasmeen J. Santos, José J. Escarce, Susan L. Ettner, Claudia Der-Martirosian, Pushpa Raja, Alexander D. McCourt, John Fortney, Emma E. McGinty

**Affiliations:** 1 Division of General Internal Medicine and Health Services Research, Department of Medicine, David Geffen School of Medicine, University of California, Los Angeles, Los Angeles, California, United States of America; 2 Center for the Study of Healthcare Innovation, Implementation & Policy, Veterans Affairs Greater Los Angeles Healthcare System, Los Angeles, California, United States of America; 3 Department of Health Policy and Management, Fielding School of Public Health, University of California, Los Angeles, Los Angeles, California, United States of America; 4 Johns Hopkins Bloomberg School of Public Health, Baltimore, Maryland, United States of America; 5 Department of Psychiatry and Behavioral Sciences, School of Medicine, University of Washington, Seattle, Washington, United States of America; 6 Health Systems Research Center of Innovation for Veteran-Centered and Value-Driven Care, VA Puget Sound Healthcare System, Seattle, Washington, United States of America; 7 Division of Health Policy and Economics, Weill Cornell Medicine, New York, New York, United States of America; PLoS ONE, UNITED STATES OF AMERICA

## Abstract

**Background:**

Until the COVID-19 pandemic, it had not been possible to examine the effect of rapid policy changes surrounding telemental health on patient-reported mental health care access, costs, symptoms, and functioning. Sizable variation in telemental health use by patient race-ethnicity, age, and rurality, and in its adoption across healthcare settings, underscores the need to study equitable dissemination and implementation of high-quality telemental health services in the real world. This protocol describes an explanatory sequential mixed-methods study that aims to examine the effects of state telemental health policy expansion on patient-reported mental health outcomes, as well as the policy-to-practice pathway from the perspectives of state leaders, clinicians, and staff who care for underserved patients.

**Methods:**

This study uses legal mapping research methods to characterize the effective dates and specific provisions of telemental health policies (e.g., Medicaid reimbursement, private payer laws, professional licensure requirements) before and during the COVID-19 pandemic in all 50 U.S. states and Washington, D.C. Then, we will examine state factors (e.g., COVID-19 cases, broadband internet access) explaining these telemental health policies using discrete-time hazard models. The primary quantitative analysis employs a difference-in-difference approach to predict effects on outcome measures using a nationally representative survey of individuals. Using the Medical Expenditure Panel Survey, we will examine policy effects on (a) access to, use of, and expenditures related to mental health care and (b) mental health outcomes, functioning, and employment. Finally, qualitative methods will be used to obtain feedback from state leaders, administrators, clinicians, and clinic staff members on how state telemental health policy expansion influenced mental health services delivery during the pandemic, with a focus on improving safety-net care. We will use a positive deviance approach to select key partners from 6 “high” and 6 “low” telehealth expansion states for interviews and focus groups.

**Discussion:**

The overall study goal is to better understand the effect of pandemic-related state policy changes around telehealth on patient-reported mental health care access, costs, symptoms, and functioning. By characterizing variations in telehealth policies and their downstream effects, this mixed-methods study aims to inform equitable dissemination, implementation, and sustainment of high-quality telemental health services.

## Introduction

The COVID-19 pandemic amplified mental health needs, especially for underserved populations. The World Health Organization reported that the pandemic prompted a 25% increase in anxiety and depression prevalence worldwide [1, 2]. Since its onset, Americans have reported elevated levels of mental health symptoms (42% of adults with anxiety/ depression), as well as suicidal thoughts (11% of adults seriously considered suicide) [3–5]. During the pandemic, more than 1 in 10 were unable to receive mental health counseling [5]. Treatment barriers include lack of providers, unaffordability, and no time off work [6].

Underserved minoritized populations are most vulnerable to COVID-19 and have been disproportionately impacted by the pandemic [6]–reporting worse mental health symptoms, higher unemployment [7], fear of job loss [8], and more unmet mental health needs than white Americans [3, 5, 9]. Mental health care access remains insufficient and fraught with challenges, especially for Medicaid patients who are often seen in safety-net clinics. Even though safety-net health systems have delivered more mental health and substance use disorder services in recent years [10], underserved patient groups still lack adequate access to timely, high-quality treatment [11]. Lack of health insurance, limited English proficiency, and inadequate community resources are treatment barriers [12]. Strategies to improve health care broadly, such as improving access to care, engagement in care, and the quality of care, could reduce mental health care disparities [13, 14]. For instance, quality improvement efforts aimed at implementing evidence-based collaborative care models in primary care settings were found to reduce depression disparities due to lower unmet need for appropriate care among Hispanic and African American patients relative to white [15]. Because depression lowers one’s ability to tend to physical health, it confers increased mortality [16] not only from suicide but mostly from chronic medical disease [17]. Thus, equitable access to mental health treatment is crucial to mitigate racial-ethnic disparities in medical care and physical health.

Telemental health, which covers telemedicine for mental health services, connects patients and providers via electronic platforms that enable video/phone visits to bridge gaps in care access widened by COVID-19. To allow for flexible healthcare delivery, the federal Public Health Emergency declaration resulted in temporary changes to telehealth policies at all governmental levels, in turn affecting all payors, especially Medicaid [18]. State governments varied in their decisions on which telemental health services will be covered by each of their Medicaid programs (e.g., reimbursement of audio-only services), as well as in leglislating policies to allow for delivery and/or payment parity of telehealth services through Private Payor laws [18]. Telehealth’s rapid expansion during the pandemic has also highlighted the digital divide, or the gap between those who have access to broadband internet, computers/smartphones, and the digital literacy required to use these devices and those who do not. A recent umbrella review of systematic reviews reported that telehealth, though an effective and acceptable form of mental health services delivery, has limited evidence on large-scale implementation across diverse settings [19]. Combining previous evidence and pandemic experiences may facilitate realistic planning for the future dissemination, implementation, and sustainment of telehealth.

The study described in this protocol provides an innovative approach to closing evidence gaps in telehealth use via naturalistic study before and during the COVID-19 pandemic. First, it will apply legal mapping research methods to study the impact of telemental health policy on mental health outcomes at a broad national scale during a critical time. We leverage variation in state telemental health policy to study impact on mental health services delivered to treat prevalent depression and anxiety disorders within community-dwelling populations, especially working age adults on Medicaid. Legal mapping research methods will allow us to characterize and then isolate policy effects, such as Medicaid’s reimbursement of audio-only services. There is potential to fill gaps in existing research based on administrative or electronic health record data for audio-only services, which were affected by coding inconsistencies during the rapid pandemic-driven expansion of telehealth. Second, this study will include nationally representative survey measures of mental health, functioning, and other patient-reported outcomes. Studies have learned much from mental health service utilization claims, but our study moves beyond analyses of service use to understand patient perceptions of care access as well as population mental health and functioning. The survey newly probes for pandemic-related challenges to mental health and mental health care, including telemental health use, nationally. Concern is warrented for worsening mental health care quality and outcomes during the pandemic, alongside rapidly redesigned mental health services infrastructure to accommodate telemental health. Thus, it is important to utilize various strategies to examine the impact of states’ telemental health response, as well as sufficiency of safety-net clinical infrastructure, in meeting population mental and other health care needs. Third, this study will leverage a pandemic to identify and learn from positive deviation (in this case, outlying states that most greatly expanded telemental health policies) about whether their safety-net and other clinics were able to improve mental health services delivery to underserved patients and the larger population. The study described in this protocol has potential to help identify patients’ specific needs and help clinics to better address major challenges in mental health care access, specifically meaningful telemental health reimbursement and regulation. Project impact will be further elevated by close and bi-directional interactions with committed national organizations (i.e., National Council for Mental Wellbeing, National Association of Community Health Centers), necessary to inform policymakers and stakeholders about the risks and benefits of relaxing those telemental health policies with the end of the Public Health Emergency declaration.

### Study aims and hypotheses

#### Aim 1

To characterize states’ telemental health policies and identify state-level determinants of expansion. We hypothesize that state telemental health expansion (i.e., policy adoption) will be predicted by high COVID-19 infections and deaths, rurality, mental health professional shortage, and broadband internet coverage.

#### Aim 2

To examine effects of state telemental health policy expansion during the COVID-19 pandemic on a) access to, use of, and expenditures related to mental health care and b) mental health outcomes, functioning, and employment. We hypothesize that state telemental health expansion (i.e., policy adoption) will a) increase (or mitigate pandemic-related reductions in) access to, use of, and expenditures on mental health care, and b) improve (or mitigate pandemic-related worsening of) mental health symptoms and functional outcomes, e.g., quality of life and employment.

#### Aim 3

To understand key partner perspectives on how state telemental health policy implementation and expansion influenced mental health services delivery during the pandemic, focusing on the impact in safety-net settings. Through interviews with state leaders, administrators, clinicians, and clinic staff, we will collect feedback on policy-to-practice telemental health implementation and impacts on mental health care quality and equity to explain Aim 1 and 2 findings about state telemental health policy approaches during the COVID-19 pandemic. We will also elicit input regarding the risks and benefits of relaxing telemental health regulations and reimbursement, especially for underserved patients.

## Methods

### Study design

This study uses explanatory sequential mixed methods [20] to understand the impact of state telehealth expansion on mental health care access, costs, symptoms, and functioning nationally. We will first analyze quantitative data and then collect qualitative data to help explain our quantitative results. Aim 1 employs legal mappintbag (“50 state survey”) to characterize telemental health policies for each state, as well as identify state-level determinants of telemental health expansion. Aim 2 uses econometric techniques to analyze the effects of the state telemental health expansion on patient care quality and mental health outcomes from the nationally representative Medical Expenditure Panel Survey (MEPS, panel data). Aim 3 brings in qualitative methods to analyze multilevel partner perspectives on how the state telemental health policy expansion influenced mental health services delivery, focusing on implementation of telemental health care in safety-net clinics (e.g., Federally Qualified Health Centers). We will rank-order states by telemental health expansion as characterized in Aim 1, to conduct in-depth interviews and focus groups among state leaders and safety-net clinics within 6 “high” and 6 “low” telemental health expansion states. Aim 3 qualitative findings will help interpret and contextualize Aim 1 and 2 quantitative findings regarding telemental health expansion impacts on mental health care and outcomes. We will then be well poised to identify organizational attributes and testable implementation strategies for equitable telemental health adoption, quality, and scale-up in pragmatic settings.

### Study period

The study period for the overarching study is 2018–2022. In Aim 1, time adoption of each telemental health policy across all 50 states will be calculated from 1/1/2018 until the end of the period of interest or policy adoption, whichever comes first. In Aim 2, Panels 23–26 (2018–2022) of the MEPS longitudinal data will be used. Aim 3 qualitative interviews and focus groups will seek to understand telemental health policy-related changes to mental health services delivery during the peri-pandemic study period, as well as probe for details on providing high-quality, equitable hybrid (in-person, video, phone, etc.) care at present.

### Data sources

#### Aim 1 legal mapping (“50 state survey”)

For Aim 1, our study team will assemble a database using legal mapping to characterize telemental health policies across all 50 U.S. states and identify state-level determinants of telemental health expansion. We will identify relevant telehealth policies (including policy provisions) through full-text searches of large legal research databases (e.g., Thomson Reuters Westlaw, Lexis Nexis). This primary data collection will involve extensive review of various state-specific legal documents including executive orders, Medicaid policy statements, session laws, and other state regulatory documents compiled on state-specific websites. Effective dates will be collected and coded as the date that the policy took effect. Policies focus on mental health care access, use, cost, and outcomes, including Medicaid reimbursement policies and private payer policies, and policies dictating requirements for providers and other health care professionals (Table 1). To ensure validity of our policy mapping data, two team members will independently find and code documents for a sample of states. We will compare the findings, identify differences in interpretation, and ensure consistency in approach before coding the remainder of the policies. Once our policy dataset is complete, we will compare our findings to any existing databases, e.g., Center for Connected Health Policy (CCHP). Should inconsistencies occur between our results and secondary data sources, we will consult the text of the relevant law and seek clarification from a legal expert on our study team. We will resolve any discrepancies through discussion with the larger research team. The constructed telemental health policy database will include variables representing telemental health policy expansion, including whether policies were adopted and the number of policies adopted, for each of the 50 states.

**Table 1:** Telehealth policy adoption expansion over time with substantial variation across states.

**State Medicaid Programs** ***(Population affected*: *Medicaid)***
Audio-only/Telephone Reimbursement
Store-and-Forward Reimbursement (e.g., eConsult)
Remote Patient Monitoring Reimbursement (e.g., app analytics)
Facility Originating Site Restrictions (e.g., telehealth to home)
Transmission & Facility Fee Reimbursement
Informed Consent Requirements for Telehealth
* Live video will not be not studied because it was reimbursed across all states before and during the pandemic.
**Private Payer Laws** **(*Population affected*: *Private insurance)***
Private Payer Laws Exist
Payment Parity Required
**Professional Regulations** ***(Population affected*: *Everyone)***
Number of Interstate Compacts per State

**Aim 2 MEPS.** Aim 2 will use the MEPS panel data. The MEPS Household Component (Panels 23–26, 2018–2022) follows and collects data from a nationally representative sample of households over time, using an overlapping panel design [21]. The Agency for Healthcare Research and Quality (AHRQ) selects a new panel of households each year, and collects data covering two years in five interview rounds (mean, 4.6 completed interviews) [22]. MEPS elicits data on sociodemographics, employment, self-rated physical and mental health, chronic and mental health conditions, insurance coverage, access to care, and health care use and expenditures. Data are arranged into longitudinal files that identify responses to each interview round, as well as the date and reason for each healthcare utilization event. Average point-in-time response rates for recent panels and the combined overall response rates for recent years are respectively 46% and 41%. Beginning 2021, the MEPS event files included indicators for whether the event was in-person or telehealth, as well as telehealth modality (phone, video, or other).

#### Aim 3 qualitative interview and focus group data

In Aim 3, interviews and focus groups will be conducted using semi-structured interview and discussion guides tailored for each group, with open-ended questions and probes as needed. Question development will be guided by Fortney et al.’s framework [23] and will include constructs that cannot be easily obtained using MEPS. Questions will focus on how three domains impact telemental health access and quality: 1) telehealth regulation at the community level, 2) telehealth implementation at the organizational level, and 3) telehealth quality at the individual level. The goal of the interviews with state leaders and focus groups with safety-net clinicians is to gather insights about what differentiates between high and low telehealth expansion states and identify context-specific strategies, as well as the impact of state telehealth policies on mental health services. State leaders will be asked about their perceptions on how state telemental health policies were implemented and the impact of these policies on the delivery of mental health services in their state. Safety-net clinic leaders, as well as clinicians/staff, will be asked about their experience providing a mix of in-person, video, telephone, and other telehealth modalities, in addition to barriers and facilitators to high-quality, equitable telemental health implementation.

The interview and focus group discussion guides will be refined based on feedback from an Advisory Board comprised of research experts and representatives from the National Council for Mental Wellbeing and National Association of Community Health Centers. The Advisory Board will provide input regarding interview and focus group design and content, as well as pilot test with cognitive interviews. The qualitative study team will conduct all interviews and focus groups virtually (e.g., phone, Zoom). Interviewees will be asked for verbal consent after review of study goals, risks, and benefits. Interviews will last approximately 30–60 minutes and focus groups will last 60–90 minutes. Participants will receive $50 and $100 incentives, respectively.

### Study sample

The Aim 1 study sample will be a cohort of all 50 U.S. states. The Aim 2 study sample will include 3 cohorts from MEPS Panels 23–26: 1) working age (19–64 years) *without* Medicaid; 2) working age *with* Medicaid; 3) seniors (65+ years). Because patterns of mental healthcare utilization, including telemental health use, differ for children, they will only be included in secondary analyses.

In Aim 3, we will apply a positive deviance approach to select 6 “high” and 6 “low” states from the top and bottom quartiles based on rank-order of telemental health policy expansion identified in Aim 1 [24, 25]. Table 2 delineates our sample recruitment and participant selection for state leader interviews and clinician focus groups. From the 12 selected states, we will interview 12 primary care and 12 mental health state leaders, with recruitment facilitated by the National Council for Mental Wellbeing and National Association of Community Health Centers. We will also conduct twelve focus groups with 4–6 safety-net clinicians and leaders, with each group representing twelve states. Chosen states will be balanced by rurality, which is disproportionately affected by the digital divide and poor mental health care access. Additional recruitment of state leaders and safety-net clinics within selected states may be considered until reaching thematic saturation.

**Table 2:** Sample recruitment and participant selection.

State Leaders (Year 1.5–2)	Clinicians and Clinic Leaders (Year 2–3)
**INTERVIEW Sampling Strategy:** Based on criterion met in prior Aim 1, not purposeful sampling.	**FOCUS GROUP Sampling Strategy:** Snowball sampling (chain-referral method through interviews with state leaders), achieves greater participation than purposeful sampling. State leaders from interviews will suggest which clinicians or clinic leaders to recruit as focus groups participants
**Inclusion Criteria:** Each state has 1 Primary Care Association leader and 1 Mental Health Commissioner.	**Individual Inclusion Criteria:** From the 12 states, we will recruit clinicians or clinic leaders with subject matter expertise in telehealth. We will prioritize inclusion of clinicians/leaders who have been working in safety-net settings since pandemic onset.
**Sample size:** From the 12 selected states, we will interview 12 primary care and 12 mental health state leaders.	**Sample size:** We will conduct 12 focus groups with 4–6 safety-net clinicians and clinic leaders representing 12 states.
**Advisors:** National Association of Community Health Centers closely partner with and maintains the directory of primary care leaders, while National Council for Mental Wellbeing does the same for mental health leaders.	

### Measures

**Fig 1:**
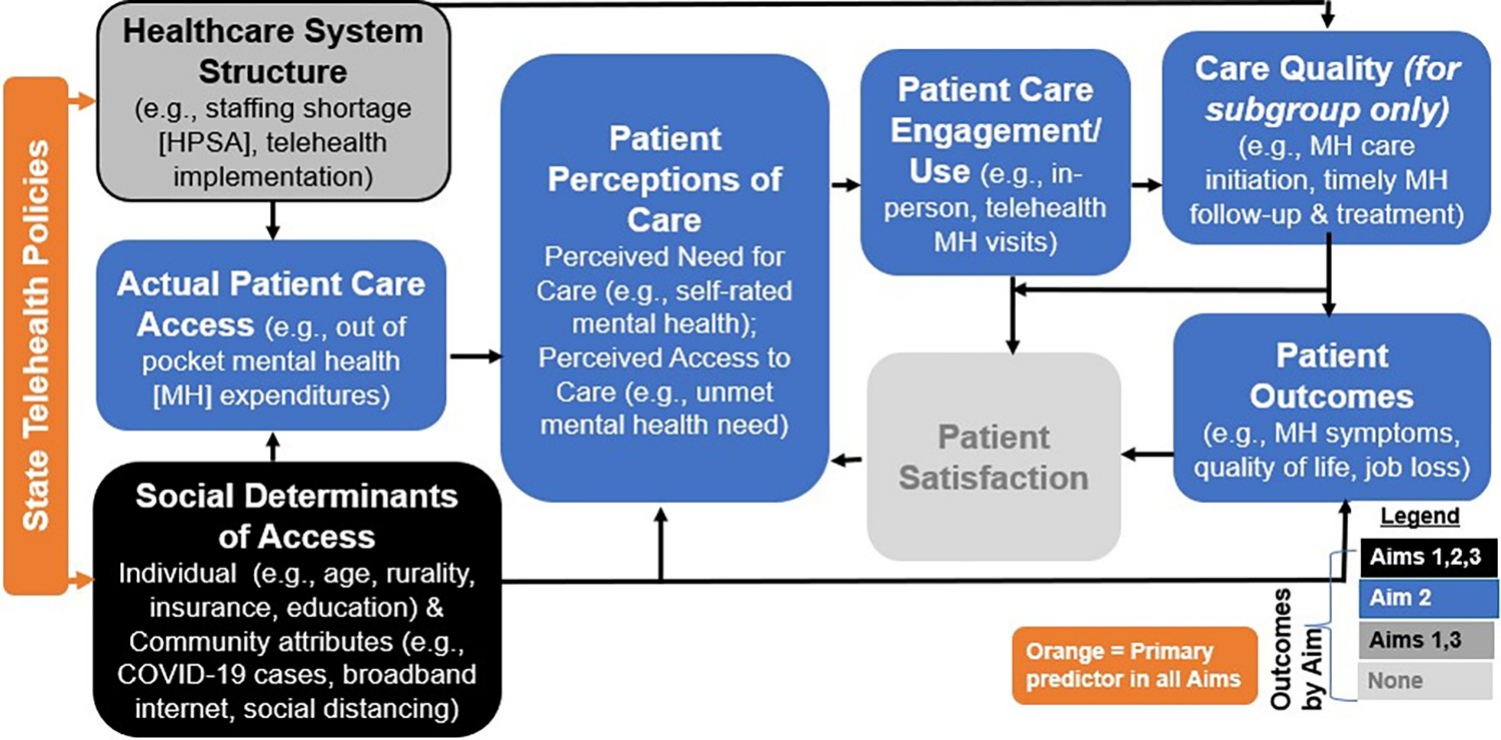
Access framework for this study adapted from Fortney et al.

We adapt Fortney et al.’s access framework [23] to guide study variable selection for both quantitative and qualitative aims (Fig 1). This framework re-conceptualizes care access and quality in the digital age by accounting for both in-person and telemental health care encounters. It considers how well the structure of the health care system (e.g., staffing, telehealth implementation) accommodates each patient’s characteristics, including their social determinants of access (e.g., age, rurality), their health status, and their community’s characteristics (e.g., broadband internet, COVID-19 cases, social distancing).

Aim 1 independent variables represent expansion of telemental health policies (e.g., audio-only reimbursement) in each of the 50 states, including binary indicators for policy adoption and the number of policies adopted. In Aim 1, contextual state-level variables from the Fortney model [23] will be adapted, including local severity of the pandemic, rural-urban status, Health Professional Shortage Areas (HPSA), and high-speed internet coverage (Fig 1). We will use binary indicators for policy adoption (e.g., audio-only reimbursement or not). Then, we will count telehealth-friendly arrangements, such as Interstate Compacts that make it easier for healthcare providers to practice in multiple states, and construct an equally weighted summary policy index measure. If there is correlation between policies, we will construct a second data-driven policy index measure using principal component analysis methods, which reduces a set of related variables into a summary measure based on the internal correlation structure of those measures.

Most Aim 2 outcome variables drawn from MEPS are point-in-time variables measured at each interview, although some have a look-back period. MEPS event files enable use/cost measure construction at temporally granular levels. Outcomes for the MEPS analyses will include mental health care access, use (e.g., mental health-related office visits, emergency visits, hospitalizations, and medications), and costs and mental health status (e.g., self-rated mental health, Kessler Index of non-specific psychological distress, Patient Health Questionnaire) and functioning (e.g., Veterans RAND 12-item health survey, job loss) (Table 3). Contextual variables include state characteristics and area characteristics by commuting zone, which are geographic areas where people live and work and may include more than one county to more accurately reflect local economies (Table 4).

**Table 3:** Aim 2 MEPS study outcomes.

**Outcomes for MEPS Analyses** *(longitudinal survey)*
**A. Mental health care access, use, costs** *(by subject-quarter)*
Amount of mental health care expenditures
No. mental health care office visits (in-person/telehealth)
No. mental health emergency visits, hospitalizations
No. mental health prescription medications
**B. Mental health status & functioning** *(by subject-interview)*
Self-rated mental health (% “Fair” and “Poor”)
Kessler Index (K6) of nonspecific psych distress
Patient Health Questionnaire-2 (PHQ-2) depression screener
Veterans RAND 12-Item (VR-12) Mental Component Score
Job loss. (MEPS enables us to ascertain when subjects lose their jobs and the reason.)

**Table 4:** Aim 2 contextual variables.

State Characteristics	Area Characteristics (by Commuting Zones)
**Telehealth policy expansion**: We will use indicators for state policy adoption (e.g., audio-only reimbursement or not). We will construct a summary policy index measure by counting the number of telehealth-friendly policies adopted.	**Local area unemployment statistics:** The U.S. Bureau of Labor Statistics produces monthly data on labor force participation, employment, and unemployment for states, counties, and metro areas (the “jobs report”).
**Pandemic-related policies:** The COVID-19 State Policy Database from Boston University, has start and stop dates for key domains of state policies, which enables us to determine which policies are in place on each day. Domains related to policies to curb spread of the virus (e.g., stay-at-home orders, school closures), as well as support policies (e.g., eviction moratoriums). Similar data bases exist, e.g., Opportunity Insights.	**Adherence to social distancing metrics**: Cuebiq, a geospatial data company, collects daily anonymized location and traffic data from millions of US mobile devices and provides their data free for academic research. Data include county-level metrics that reflect adherence to social distancing (e.g., percent of devices staying home and going to work). Metrics were found to be affected by stay-at-home orders and affect viral transmission.
**COVID-19 vaccination:** We will use daily state-level data on cumulative COVID-19 cases, death (per 100k persons), and vaccination rates (total doses given, people with 1+ doses, people with 2 doses) collected and archived by the New York Times using governmental and health department data, e.g., Centers for Disease Control.	**County policies:** The COVID-19 State and County Policy Orders Database from Department of Health & Human Services, reports start and stop dates for county-level policies on stay-at-home orders, business closures, etc. It contains useful information for 790 of 3,142 large urban counties that may have diverged from state policies.
**Others** (including, but not limited to): Medicaid expansion (and/or state payor mix) from Kaiser Family Foundation data; party affiliation of governor and/or legislature	**Additional area characteristics:** Includes local severity of the pandemic (e.g., COVID-19 cases/deaths), rural-urban status (from Area Health Resources File), Mental Health Professional Shortage Areas (HPSA), high-speed internet coverage (as per Federal Communications Commission).

Aim 3 qualitative interviews will characterize key partner perspectives from 12 states (6 high- and 6 low-adoption states as rank-ordered in Aim 1) on how state telemental health policy influenced mental health services delivery during the pandemic, focusing on safety-net care. Interviews and focus groups will be used to elicit themes regarding telemental health policy implementation during the COVID-19 pandemic and impacts on care quality and equity from both state leader and frontline clinical perspectives.

### Analysis

#### Aim 1 survival analysis

To estimate the number of months until adoption of each telemental health policy across all 50 states between 2018–2022, we will conduct discrete-time hazard models [26] (survival analysis) by calculating logit-hazards monthly with 95% confidence intervals. Time to adoption will be calculated from 1/1/2018 until telemental health policy adoption or until the end of the period of interest (i.e., censored data), whichever comes first. We will incorporate *a priori* state-level covariates (Table 4; e.g., state demographics and payor mix) into all models to examine the influence of these variables on state telemental health expansion. To understand the impact of pandemic onset, we will include a fixed effect for time using a post-March 2020 indicator. We will also include the time-varying predictor of COVID-19 cases/deaths to capture state pandemic severity. Sensitivity analyses will be performed to investigate potential residual confounding by modeling time in varying segments, as well as lagging COVID-19 cases/deaths by varying number of months. Additionally, states that expanded Medicaid programs may rely on telehealth to meet the needs of newly insured disadvantaged populations, so our sensitivity analyses will examine for associations between telemental health policy adoption and state Medicaid expansion using Kaiser Family Foundation’s database [27]. Finally, we will examine for associations between state summary policy index and contextual variables using count data regression models with state random effects. Based on the 50 state sample, we should have 80% power to detect hazard ratios for covariates in the Cox models of at least 2.33 assuming a 0.05 two-sided level of significance. This minimally detectable hazard ratio is based on a general, standardized covariate.

#### Aim 2 difference-in-differences study

To compare trends in outcomes among those living in states before and after expansion of state telemental health policies, a staggered difference-in-differnces approach will be used [28]. We will analyze each of three cohorts separately. For individual policy outcomes, analyses will be conducted for the affected population (e.g., Medicaid for audio-only reimbursement).

MEPS data enable us to compare changes over time in outcomes for specific individuals and, therefore, to control for time-invariant unobserved characteristics that may be correlated with pandemic effects. The regression model for our MEPS analyses is: $Y_{itcs}=\alpha_{i}+\beta {TH}_{ts}+{\mu I}_{t}^{post}+\varphi I_{t}^{post*}{TH}_{ts}+h{MH}_{i0}*{Trend}_{u(i)}+\gamma X_{it}+\theta P_{ts}+\sigma V_{ts}+\tau Z_{tc}+\varepsilon_{itcs}$

***Y_itcs_*** is an outcome for subject *i* residing in commuting zone *c* and state *s* interviewed at time *t* (refers to calendar year for utilization/cost outcomes, and to interview round for mental health/functioning outcomes);

***α_i_*** are individual fixed effects, which account for time-invariant individual characteristics;

***TH_ts_*** is an indicator variable equal to “1” if state *s* had a specified telemental health policy in effect during time *t*; alternatively, it is a count variable equal to the number of telemental health policies in effect at state *s* during time *t;*

$I_{t}^{post}$ is an indicator variable with the value “1” in the post-pandemic period and “0” otherwise. $I_{t}^{post}$ is interacted with ***T_ts_*** to estimate a separate coefficient, ***φ***, for states that adopted telemental health policy during the pandemic;

***Trend_u(i)_*** is a linear trend in the time, *u(i)*, since subject *i*’s first MEPS interview; and an interaction term with *Trend_u(i)_* to allow for subject *i*’s underlying outcome trajectories to differ depending on baseline self-rated mental health, ***MH*_*i*0_.** (Baseline values are from the first interview.) Accounting for heterogeneity in trajectories will minimize the bias in the estimates of telemental health expansion effects that could occur if mental health at baseline is unequally distributed between states before and after expansion of telemental health.

***X_it_***, the vector of individual characteristics, is limited because individual fixed effects absorb the effects of time-invariant individual characteristics. We still include characteristics that change over time and are not expected to be secondary to pandemic effects (e.g., marital status).

***P_ts_*** is a vector of pandemic-related policies in effect in state *s* at time *t*, ***V_ts_*** is the vaccination rate in state *s* at time *t*, and ***Z_c_*** is a vector of additional commuting zone-level covariates including broadband internet availability, social distancing, etc. ***ε_itcs_*** is the error term. The Greek symbols are coefficients to be estimated.

When the treatment (telemental health policy) is implemented in staggered fashion, Callaway and Sant’Anna [28] have shown that the simple difference-in-differences estimator may be biased. In this case, the simple estimator is the weighted average of the treatment effects at each of the implementation times. Callaway and Sant’Anna developed an approach to test and deal with this problem. In our study, however, we address it through model specification using a post-March 2020 indicator variable, that is, we allow for different policy effects before and after pandemic onset. We will also develop a scheme for putting policies into the regression model one, two, three at a time and so forth to figure out which have big effects on mental health outcomes and examine for policy correlation using the variance inflation factor.

We will use linear models, including linear probability models for binary outcomes, to facilitate direct interpretation of the coefficients and avoid the implicit interactions present in nonlinear models [27, 29]. Since fixed effects exist at the level of the unit of analysis, we cannot estimate nonlinear models for MEPS in particular. Nonetheless, linear probability models yield reliable estimates of mean effects [30] and are widely used [21, 22, 31]. Since the error terms in these models are heteroskedastic of known form, we will use heteroskedasticity-consistent variance estimators. We will examine the intra-class correlation (ICC) coefficient to assess magnitude of dependence at the state levels and ensure appropriate accounting for clustering. To account for the sampling design of MEPS, including weighting and clustering, we will perform all analyses using Stata survey commands.

Furthermore, we will conduct subgroup analyses and compare to main analyses. We will stratify by age group (including seniors and adolescents aged 10–17 years) for the general population, for men and women, and for underserved subgroups where the effects of telemental health expansion are likely concentrated, including (1) persons with high school or no degree (28% of the U.S. population) [32]; (2) African Americans and Hispanic Americans (~31% of total) [33]; (3) those living in rural areas (~14% of the U.S. population); and (4) those with probable mental illness and a new episode of mental health care [13, 34, 35]. For subgroups 1–3, we will estimate the effects of telemental health expansion by estimating separate models. For subgroup 4, we will examine process measures of care quality—that is, treatment initiation, at least minimally adequate follow-up, and need for acute psychiatric care as done in previous research [13, 34, 35]. Statistical power to detect significant interactions in these subgroup will be limited, however. Our sensitivity analyses will also estimate probit/logit models and compare the marginal effects by averaging the predicted margins over the whole sample. In doing so, we will need to exclude fixed effects for MEPS panel data.

We estimated numbers for the smallest cohort of working-age Medicaid adults, assuming that we will have greater power to detect differences for larger cohorts (Table 3). With MEPS data, we will be able to detect differences of ~3% (α = 0.05) or ~4% (α = 0.01) for outcomes (e.g., baseline 17% with mental health expenditures [36]), but this should be a conservative estimate given that we will have repeated observations for subjects observed in these data. The Chi-Square test is a conservative simplification of the regression modeling approach that will be used for the planned analysis. In our preliminary analysis, we identified effects of a similar magnitude, suggesting that it is reasonable to expect that we will have a sufficiently large sample to detect policy effects on outcomes.

#### Aim 3 qualitative analysis

Two qualitative analytical approaches will be used. The first approach will include rapid qualitative analysis methods [37, 38], which has been shown to save time and reduce costs [39] while yielding valid results like those from traditional coding [37]. For the rapid analysis approach, a templated summary table of key domains based on the interview or focus group guide will be developed. All transcripts will be divided and independently summarized by study team members. This initial rapid analysis will also identify additional/emerged domains. Then, senior researchers on our study team will randomly select interviews and focus groups to conduct secondary reviews of summaries and discuss discrepancies with the team to ensure consistency. All summaries will then be consolidated into high-level summary documents to identify key points and commonly occurring themes across all focus groups. We will also examine interview and focus group summaries grouped by type (e.g., high telemental health/urban, high telemental health/rural, low telemental health/urban, low telemental health/rural). The second approach will include a thematic analysis using both inductive and deductive methods to allow coding and theme/sub-theme development [40]. This process will expand on what we learn from the initial rapid analysis. Our research team will jointly develop the list of codes, using both an inductive “grounded theory” development of codes and a deductive coding based on the study theoretical framework (*a priori* codes). An audit trail will be used to document all qualitative methodological decisions. To ensure quantitative findings inform the qualitative approach, we will share relevant Aim 1 and Aim 2 state findings and ask for interviewee feedback. For example, we may describe which telemental health policies were in effect based on Aim 1 and share the percent of individuals reporting telemental health use over past month derived from Aim 2. We will use Atlas.ti (v.9+) to organize data analysis.

In accordance with the Belmont Report and under the authority of the Common Rule, the UCLA Office of the Human Research Protection Program (OHRPP) determined that our quantitative project does not meet the definition of human subjects research (IRB#22–001956) and our qualitative study meets the criteria for an exemption from IRB review (IRB#22–001993).

## Discussion

Until the COVID-19 pandemic, it had not been possible to examine the implementation and the effects of rapid policy changes surrounding telemental health on patient-reported mental health care access, quality, costs, symptoms, and functioning. The pandemic has negatively impacted population mental health, to which low-income, racial ethnic minorities have been especially vulnerable. Telemental health is an effective tool used for various mental health conditions and has potential to close care access gaps across patient groups (for rural and for racial-ethnic minorities). Observational research remains necessary to ensure that clinical trial results regarding telemental health effectiveness generalize to real-world patient populations [41, 42]. Previous studies largely focused on analyzing administrative claims [43, 44], with limited data on direct policy impact and patient perspective. In this study, Aims 1 and 2 will examine effects of state telemental health policy expansion on patient-reported mental health outcomes.

Our study will secondarily close evidence gaps on the policy-to-practice pathway with input from state leaders, clinicians, and staff who care for underserved patients. Telemental health policies have rapidly expanded in an attempt to bridge access gaps by income and race-ethnicity widened by COVID-19 [45], with little knowledge about the extent of their adoption and impact across safety-net primary care and mental health clinics nationally. We strive to study equitable implementation of telemental health services, as well as the mix of telehealth and in-person services delivered to safety-net patients at scale. The COVID-19 pandemic allows us, for the first time, to leverage a naturalistic opportunity to improve telemental health services focusing on underserved populations.

Aim 3 qualitative research will provide a basis for identifying organizational attributes and testable implementation strategies for equitable telemental health adoption, quality, and scale-up in pragmatic settings. Guided by Fortney et al.’s access framework [23], we will garner multilevel stakeholder perceptions of how state telemental health policy expansion influenced mental health services delivery during the pandemic, with a focus on improving safety-net care. For example, if audio-only reimbursement is found to reduce unmet mental health need for underserved groups, partners can guide our research dissemination to policymakers and relevant stakeholders before temporary telemental health legislation is set to expire. If safety-net clinicians still hesitate to offer telemental health services and require in-person visits from underserved groups despite state telemental health expansion, our research will uncover reasons and provide a basis for testing implementation strategies for telemental health in future studies.

In addition to its strengths, this study has some notable limitations. First, our estimates of the causal effects of telemental health expansion may be biased if unmeasured state attributes that are correlated with the study outcomes differ between telemental health expansion and non-expansion states. Aim 1 will examine observable confounders and Aim 2 models will include individual fixed effects, as well as time-varying contextual variables, both of which substantially mitigate this concern. Second, measurement of patient-reported telemental health use from survey data sources is challenged by the availability of data only after pandemic onset. Nonetheless, variation across states in adoption of telemental health policies during this short period was substantial and thus mitigates the risk of effect non-detection (see Table 1). Although the survey excludes people who live in institutional settings, findings do apply to persons living in the community, where prevalence and burden of depression and anxiety are still high.

The Public Health Emergency declaration resulted in flexible health care delivery through temporary and permanent changes to state telemental health policies. While we know that mental health care delivery became more flexible, we need to establish how this flexibility affected actual patient care and mental health outcomes. Our study uses a mixed-methods approach to address pandemic-related challenges to mental health and mental health care in all 50 U.S. states, as telemental health’s rapid expansion during the pandemic has highlighted the digital divide. The study described in this protocol (1) applies legal mapping research methods to study the impact of telemental health policy on mental health outcomes at a broad national scale during a critical time, (2) includes nationally representative survey measures of mental health care use, functioning, and other patient-reported outcomes (e.g., Patient Health Questionnaire), and (3) leverages a natural experiment to identify and learn from positive deviant states about whether their safety-net and other clinics were able to implement telemental health and improve mental health services delivery to underserved patients and the larger population. This study will enable realistic planning for the future dissemination, implementation, and sustainment of telemental health and has potential to help identify patients’ specific needs and help clinics to better address major challenges in mental health care access, specifically meaningful telemental health reimbursement and regulation.

## List of abbreviations

AHRQ: Agency for Healthcare Research and Quality
CCHP: Center for Connected Health Policy
COVID-19: Corona Virus Disease 2019
HIPAA: Health Insurance Portability and Accountability Act
HPSA: Health Professional Shortage Areas
ICC: Intra-Class Correlation
MEPS: Medical Expenditure Panel Survey
RDC: Research Data Center
